# Health insurance literacy in the Netherlands: The translation and validation of the United States’ Health Insurance Literacy Measure (HILM)

**DOI:** 10.1371/journal.pone.0273996

**Published:** 2022-09-01

**Authors:** Laurens Holst, Aafke Victoor, Anne Brabers, Jany Rademakers, Judith de Jong

**Affiliations:** 1 Nivel, The Netherlands Institute for Health Services Research, Utrecht, The Netherlands; 2 CAPHRI, Maastricht University, Maastricht, The Netherlands; PLOS: Public Library of Science, UNITED KINGDOM

## Abstract

An important criterion for the proper functioning of a managed competition healthcare system, such as operates in the Netherlands, is that all citizens can make well-informed decisions regarding their health insurance policy. In order to achieve this, citizens need certain health insurance literacy skills. It is not known how far citizens in the Netherlands have these skills. The aim of this study is to provide a reliable instrument that measures the health insurance literacy of citizens in the Netherlands. It would be based upon an existing instrument developed in the US. We translated the US Health Insurance Literacy Measure (HILM) into Dutch. Furthermore, we established the psychometric properties of the Dutch version (HILM-NL), and we validated the HILM-NL in a general population sample by sending surveys to members of the Nivel Dutch Health Care Consumer Panel (DHCCP) in February and March 2020. The response rates were respectively 54% (n = 806) and 56% (n = 595). No insurmountable difficulties with equivalence were encountered throughout the translation process. The HILM-NL showed an acceptable level of internal consistency, good test-retest reliability, and a good construct validity. The HILM-NL is a reliable instrument for measuring health insurance literacy among citizens in the Netherlands. With this instrument, it is better possible both to assess how these citizens choose and use a health insurance policy, and also the difficulties they face. It enables citizens in the Netherlands to be supported better in making well-informed decisions on health insurance.

## Introduction

Various healthcare systems including those in Germany, Switzerland, and the US have elements of managed competition [1]. Such systems require citizens to be able to choose between multiple health insurance policies from different insurers [2, 3]. Managed competition is based on the idea that citizens will switch to another insurer if this insurer offers a more suitable policy. In this way, insurers are, it is hoped, stimulated to continue offering high quality care and services within their insurance policies for a competitive price. In 2006, the Netherlands introduced a managed competition healthcare system [4, 5]. It became compulsory for all citizens living or working in the Netherlands to take out a basic insurance policy. Furthermore, all citizens were, from that moment on, expected to behave like critical consumers, asking themselves whether their current insurance policy still fulfils their needs and preferences [6]. If not, or if another insurer has a better offer, they have the option, during the yearly switching period from mid-November until the end of January, to switch to another policy from a different insurer. Health insurers, in turn, are obliged to accept all citizens for their basic insurance policies.

In order for managed competition to function in the Dutch healthcare system as intended, it is important that all citizens can make well-informed decisions regarding their health insurance policy. In 2019, citizens in the Netherlands could choose between 59 basic health insurance policies offered by 24 different insurance companies [7]. In recent years, several initiatives have been launched in order to support citizens in choosing a suitable policy. Initiatives such as “My policy profile” (mijn Polisprofiel) and the “Health insurance card” (de Zorgverzekeringskaart) have been developed in order to inform citizens better about the characteristics of health insurance policies. These initiatives should provide citizens with a more complete overview of all the different insurance policies available. However, more than half of Dutch citizens report that it is difficult to compare health insurance policies [8]. Furthermore, a large number consider it difficult to assess health insurance information on reliability, relevance, and completeness [8]. It is clear that citizens need certain skills in order to understand health insurance information.

In general, people need health literacy skills to be able to obtain, understand, appraise, and apply health care information [9]. Specifically in the context of health insurance, the concept of health insurance literacy has been developed in the United States (US). It is defined as *the extent to which consumers can make informed purchase and use decisions regarding health insurance* [10]. Similarly to the Netherlands, there were indications that citizens in the US struggle to understand the health insurance market [11]. Research has shown that many US citizens did not seem well enough prepared to make an informed decision about a suitable health insurance policy [12]. As such, recognition has grown that citizens need assistance in obtaining an insurance policy. Quincy et al. (2012) concluded that consumers have serious difficulties understanding and using health insurance. They also emphasized that there is a dearth of usable information on the precise barriers facing consumers [13]. A greater understanding of the health insurance literacy of citizens is needed, therefore, to support citizens in acquiring information about health insurance better.

In 2014, the Health Insurance Literacy Measure (HILM) was developed in the US [14]. It is a self-assessment measure of citizens’ ability to select and use a private health insurance policy [14]. It provides insight into which groups of citizens need additional support when enrolling in a health insurance policy or when using policy benefits to pay for health services once enrolled. Furthermore, it provides insight into what are the components of choosing and using an insurance policy which are most troublesome for citizens. The HILM has been developed for the entire US population and is therefore particularly suitable for being able to distinguish between citizens with the most common health insurance literacy scores (the middle range) and to a lesser extent between individual with extremely high or low health insurance literacy scores [14]. The HILM has been shown to be a reliable and validated instrument for measuring health insurance literacy [14]. In fact, we believe, it is the only one.

The HILM appears useful in providing insight into the health insurance literacy of US citizens. In order to gain a similar insight in the Netherlands, we decided to translate and validate the instrument for the Dutch situation. Therefore, the aim of this study is to:

1. Translate the United States’ HILM into Dutch.
2. Establish the psychometric properties of the Dutch version HILM-NL.
3. Validate the HILM-NL in a general population sample.

The HILM-NL has the potential to contribute to a better understanding, both of how citizens in the Netherlands choose and use a health insurance policy, and the difficulties they face. In this way, information about health insurance can be better tailored to citizens’ level. The ultimate goal is to provide better support for citizens in the Netherlands in making well-informed decisions regarding health insurance.

## Material and methods

### Health Insurance Literacy Measure

The original Health Insurance Literacy Measure (HILM) was developed by the American Institutes for Research (AIR). It consists of 21 questions that are categorised by concepts into four subscales. These are: 1) confidence in choosing a health insurance policy (six questions); 2) behaviour in choosing a health insurance policy (seven questions); 3) confidence in using a health insurance policy (four questions); and, 4) behaviour in using a health insurance policy (four questions). The four subscales can be grouped into two domains, “confidence” (subscale 1 and 3), and “behaviour” (subscale 2 and 4). Respondents were excluded if they answered fewer than three questions on subscale 1 and 2, or fewer than two on subscale 3 and 4. Answers to the questions of the domain “confidence” were scored on four-point ordinal scales. These are: not at all confident (1); slightly confident (2); moderately confident (3); and, very confident (4). Answers to the questions of the domain “behaviour” were scored on four-point ordinal scales. These are: not at all likely (1); somewhat likely (2); moderately likely (3); and, very likely (4). The mean scores per domain were then calculated on the basis of these categories. These mean scores range from one (marked one on all questions) to four (marked four on all questions). High scores imply a higher self-assessed ability in selecting and using health insurance.

### Translation and adaptation process

The translation and adaption, or cultural validation, process was carried out in 2019 by Nivel, the Netherlands Institute for Health Services Research. This adhered to the guidelines of the World Health Organization (WHO) for the translation and adaption of instruments [15]. This process consisted of four consecutive steps. These were: a forward translation; the use of an expert panel; a translation back to the original English language; and, lastly, cognitive interviewing.

Two independent Dutch translators performed the forward translation. In collaboration with the translators, the research team then discussed and resolved discrepancies between the two translations and a single Dutch draft version of the HILM-NL was agreed. This Dutch draft version of the HILM-NL was then reviewed and discussed by the research team and experts from the field. These included representatives from the Dutch Ministry of Health, Welfare and Sport (VWS), the Dutch Healthcare Authority (NZa), Zorgverzekeraars Nederland (ZN), the umbrella organisation of ten Dutch health insurers, the Netherlands Patients Federation, and the Dutch Consumers Association. The aim was to assess whether the draft version fits with the Dutch healthcare system and would be understood by all citizens in the Netherlands. Minor adjustments were then made based on the expert panel’s suggestions. The Dutch draft version was then translated back into English by a independent translator whose native language is English. There were only a few minor textual discrepancies between the backward translation and the original translation, which led to the conclusion that the HILM-NL still matches the original HILM well. Finally, cognitive interviews were conducted to investigate whether the questions in the HILM-NL were understood in the same manner by people with different background characteristics [16]. A sample of 1,000 members of the Dutch Health Care Consumer Panel were approached, online, to participate in a face-to-face interview. Forty-six panel members indicated that they wanted to participate. Ultimately, ten participants, five men and five women with a mean age 52 (range 30 to 85 years), were selected from this group, aiming for diversity in background characteristics such as sex, age, and education. The interviewer (Aafke Victoor or Laurens Holst) asked the participants to fill in HILM-NL using the so-called "Think Aloud" method in which the participants were asked to verbalise thoughts that emerge as a task is being completed [16]. This involved taking into consideration their interpretations and thoughts on the questions and the categories of answers. The cognitive interviews did not lead to major alterations in the HILM-NL, though some questions were slightly re-phrased. The final version of the instrument (HILM-NL) can be found on the Nivel website: https://www.nivel.nl/nl/publicatie/health-insurance-literacy-measurement-nederlands-hilm-nl

### Panel

Two surveys were conducted among members of the Dutch Health Care Consumer Panel (DHCCP) in February and March 2020. These were intended to calculate the internal consistency of the HILM-NL and to examine its reliability and construct validity. The DHCCP is managed by Nivel. It is a so-called access panel [17]. At the time of this study it consisted of approximately 11,000 people, aged 18 and older, who have agreed to answer questions on a regular basis related to health care experiences, opinions and knowledge. The background characteristics of these people, such as their age, level of education, income, and self-reported general health, are known. There is no possibility of people signing up for the panel on their own initiative. It is renewed on regular base to ensure that representative samples of the Dutch population can continue to be drawn.

The data are analysed anonymously, and processed according to the panel’s privacy policy, which complies with the General Data Protection Regulation (GDPR). According to Dutch legislation, neither obtaining informed consent nor approval by a medical ethics committee is obligatory for carrying out research using the panel [18]. Participation is voluntary and members are not forced to participate in surveys. They can stop their membership at any time without giving a reason.

### Samples

For this study, 1,500 members of the DHCCP were approached in February 2020, online or on paper—a mixed-mode methodology—according to their own preferences. The sample was representative of the Dutch population aged 18 and older, regarding sex and age. The HILM-NL was included within Nivel’s annual monitor, “switching health insurer”, which examines, among other things, the number of citizens who switched insurer and their reasons for switching. Panel members could skip a question if they could not, or did not want to, answer that specific one.

To examine the test-retest reliability, all panel members who received the survey in February online (1,053 of the 1,500) received the HILM-NL questions again approximately one week after closing the first survey (March 2020).

### Statistical analyses

STATA 15.0 was used to calculate the internal consistency and to examine the reliability and construct validity of the HILM-NL.

#### Internal consistency

The internal consistency of the HILM-NL, as well as that of its four subscales and two domains, were assessed by calculating Cronbach’s alphas. This statistical test measures whether several items that propose to measure the same general construct produce similar scores. In general, values of alpha ranging from 0.7 to 0.95 are considered acceptable [19]. Additionally, to reduce further the length of the HILM-NL survey, average inter-item correlations were calculated to detect redundancy. Average inter-item correlation ideally should be between 0.2 and 0.5 [20].

#### Test-retest reliability

Test-retest reliability of the HILM-NL in total, plus its four subscales and two domains, were assessed by calculating intraclass correlation coefficients (ICC). Furthermore, mean differences between the first and second HILM-NL scores were calculated. ICC values > 0.9 are considered to indicate excellent test-retest reliability, values ranging from 0.75 to 0.9 as good, 0.5 to 0.75 as moderate, and values < 0.5 as poor reliability [21].

#### Construct validity

In order to examine the construct validity of the HILM-NL, we invesigated both convergent validity (the relationship between our results and the results of a similar study) and group validity (the ability of a test to discriminate between groups).

For convergent validity, the association between the HILM-NL and the European Health Literacy Survey (HLS-EU-16) score was examined. The HLS-EU-16 focuses on the skills to obtain, understand, appraise and apply health care information [9]. Both the HILM-NL and the HLS-EU-16 are self-assessment measuring instruments. Both instruments are assumed to measure corresponding concepts, for example, the knowledge about health services and one’s own health status on one hand, and the ability to use this information to make decisions on the other [14]. It was hypothesised that *lower health literacy would be associated with lower health insurance literacy*, *and vice versa*, *higher health literacy with higher health insurance literacy*. This hypothesis was examined by comparing the mean HILM-NL scores between people with inadequate, limited, and sufficient health literacy based on the HLS-EU-16. HLS-EU-16 scores were available for part of the February sample (n = 437) from a previous study in September 2019. In this sample of people’s health literacy, which followed the scoring instructions of Vandenbosch et al. [22], 6% had inadequate, 21% limited, and 73% sufficient health literacy skills.

For group validity, a choice was made to compare, based on their use of information resources, the mean HILM-NL scores from the first survey in February of four groups of respondents. The results from the February survey provided insight into the group behaviour during the 2019/2020 switching period. Group A did not consult health insurance information, Group B consulted one source, Group C consulted two sources, and Group D consulted three or more sources, respectively. Respondents were able to choose from a list of fifteen information sources. This question was part of the same annual monitor “switching health insurer” that also included the HILM-NL. It was expected that *a higher number of sources of health insurance information consulted would be related to higher mean HILM-NL scores*. The construct validity of the HILM-NL was assessed by using ANOVA, as with the validation of the HILM (the original instrument from the US).

## Results

### Translate the United States’ HILM into Dutch

The full translation and adaption process is described in detail in a Dutch report [23], which is published exclusively on the Nivel website. One example of a point of discussion that emerged during the translation process was related to differences between the Dutch and the US healthcare systems. Citizens in the US can be insured through their employer. This means that some of the citizens in the US do not have to choose, actively, a health insurance policy themselves. In the Netherlands however, all citizens must, themselves, take out a health insurance policy. The employer is mentioned in some questions from the original US HILM. These parts are not taken into account in the Dutch HILM-NL version. In addition, during the expert panel meeting there was a discussion on how questions should be formulated for citizens in the Netherlands. The cognitive interviews showed that a number of questions should be asked more directly and simply. It became clear that citizens in the Netherlands want to be questioned in a targeted manner, without too much repetition. Consecutive questions with some repetition are, therefore, not written out completely in the Dutch HILM-NL version.

Eventually, the backward translation, together with the final version of the HILM-NL, was sent to the American Institutes for Research (AIR). The Institute approved both translations and gave permission to use, distribute, and reproduce the HILM-NL (CC BY 4.0). An electronic version of the HILM-NL can be obtained from the authors, free of charge.

### Establish the psychometric properties of the Dutch version HILM-NL

#### Respondents

Table 1 shows the composition of the members of the DHCCP who responded to the survey in February and March. The response rate in February was 54% (n = 806) and in March 56% (n = 595). The male / female ratio of respondents is almost evenly distributed in both groups. The average age of the respondents in February is 58 years; in March 55 years.

**Table 1:** Composition of the respondents of the Dutch Health Care Consumer Panel in February and March.

		DHCCP in February (n = 806)	DHCCP in March (n = 595)
Sex	Male	50%	49%
	Female	50%	51%
Age	18–39	16%	20%
	40–64	54%	55%
	65 and older	30%	25%

#### Mean HILM-NL scores in February

Twenty-five respondents to the February survey were excluded because they completed too few questions from the HILM-NL. In total, the mean HILM-NL scores of 781 respondents were calculated (Table 2). The mean HILM-NL score among the respondents is 2.62; 2.65 on the domain “confidence”, and 2.58 on the domain “behaviour".

**Table 2:** The mean score of the HILM-NL and its subscales and domains among the respondents of February 2020 (n = 781).

	Mean score	SD
HILM-NL	2.62	0.62
Subscale 1: Confidence in choosing a health insurance policy	2.74	0.63
Subscale 2: Behaviour in choosing a health insurance policy	2.56	0.79
Subscale 3: Confidence in using a health insurance policy	2.57	0.76
Subscale 4: Behaviour in using a health insurance policy	2.60	0.82
Domain 1: Confidence (subscale 1 and 3)	2.65	0.64
Domain 2: Behaviour (subscale 2 and 4)	2.58	0.69

### Validate the HILM-NL in a general population panel

The validation of the HILM-NL was examined by calculating the internal consistency, test-retest reliability, and construct validity.

#### Internal consistency

The internal consistency of the HILM-NL, as well as of its four subscales and two domains, which are all measured as Cronbach’s alphas, are shown in Table 3. The total value of alpha for the HILM-NL was 0.94, indicating that the level of internal consistency of the HILM-NL is acceptable. Each individual subscale or domain also showed an acceptable level of internal consistency, ranging from 0.78 to 0.91. The average inter-item correlations are, more or less, within the acceptable range of 0.2–0.5. The inter-item correlation of subscale 2 and 3 are slightly higher (0.60). However there are no signs of redundancy here (inter-item correlation > 0.7).

**Table 3:** Cronbach’s alphas and average inter-item correlation of the HILM-NL and its subscales and domains in February 2020 (n = 781).

	Cronbach’s alphas	Average inter-item correlation
HILM-NL	0.94	0.42
Subscale 1: Confidence in choosing a health insurance policy	0.84	0.47
Subscale 2: Behaviour in choosing a health insurance policy	0.91	0.60
Subscale 3: Confidence in using a health insurance policy	0.86	0.60
Subscale 4: Behaviour in using a health insurance policy	0.78	0.47
Domain 1: Confidence (subscale 1 and 3)	0.90	0.48
Domain 2: Behaviour (subscale 2 and 4)	0.89	0.43

#### Test-retest reliability

The intraclass correlation coefficients (ICCs), characterizing the test-retest reliability of the HILM-NL, and of its four subscales and two domains, are shown in Table 4. In total, 449 panel members responded to the HILM-NL questions both in February and March. The total intraclass correlation of the HILM-NL was 0.80, indicating that the test-retest reliability is good. Each individual subscale or domain also showed a moderate or a good level of test-retest reliability, ranging from 0.62 to 0.80. Furthermore, mean differences between the subscales and domains of the first measurement and the second measurement are small (≤0.05). This suggests too that test-retest reliability is acceptable.

**Table 4:** Intraclass correlation coefficients and mean differences of the HILM-NL and its subscales and domains (n = 449).

	ICC	Mean difference
HILM-NL	0.80	0.00
Subscale 1: Confidence in choosing a health insurance policy	0.75	-0.00
Subscale 2: Behaviour in choosing a health insurance policy	0.68	0.03
Subscale 3: Confidence in using a health insurance policy	0.73	0.02
Subscale 4: Behaviour in using a health insurance policy	0.62	-0.05
Domain 1: Confidence (subscale 1 and 3)	0.80	0.01
Domain 2: Behaviour (subscale 2 and 4)	0.71	-0.01

#### Construct validity

The outcomes of the convergent and group validity assessments can be gathered from Table 5. Both assessments were in the direction expected and our two hypotheses were confirmed as significant (p < 0.001). For convergent validity, respondents with higher levels of health literacy were associated with higher scores of health insurance literacy, and vice versa. For group validity, respondents who indicated that they have consulted more sources of health insurance information were associated with higher scores of health insurance literacy, and vice versa.

**Table 5:** The association between the mean HILM-NL scores and HLS-EU-16 levels, and the number of self-reported sources of health insurance information consulted.

		n	Mean HILM-NL score	ANOVA F (df)
Health Literacy (HLS-EU-16)	Sufficient	321	2.72	12.84* (2)
	Limited	90	2.50	
	Inadequate	26	2.20	
Number of consulted sources of health insurance information	None	109	2.36	13.01* (3)
	1 source	109	2.71	
	2 sources	91	2.73	
	3 sources or more	122	2.80	

* = P < 0.001

## Discussion

The HILM-NL is a reliable instrument for measuring health insurance literacy among Dutch citizens. No insurmountable difficulties with equivalence were encountered throughout the translation process, which led to the conclusion that the Dutch translation was satisfactory. The level of internal consistency is acceptable (0.94), as well as the inter-item correlations. The inter-item correlation of subscale 2 and 3 (both 0.6) were slightly higher than the acceptable range (0.2–0.5). This suggests that there is still room to reduce the number of questions in the survey as some may overlap.

Furthermore, the HILM-NL showed good test-retest reliability (0.8). When looking more specifically at the four subscales of the HILM-NL, however, it stands out that the test-retest reliability of subscale 2 (0.68) and 4 (0.62) can be considered as moderate (range 0.5 to 0.75). These two subscales show a relatively greater deviation (≥0.79) between the mean scores of the two tests, compared to the other two subscales (≤0.76). The test-retest reliability of the HILM-NL could show greater deviation in the further validation of other Dutch samples. Based on our results, we recommend re-examining the questions that focus on the behaviour of citizens when choosing and using a health insurance policy to determine if the questions can be interpreted in multiple ways.

The construct validity of the HILM-NL was investigated by measuring two hypotheses. Firstly, that lower health literacy would be associated with a lower health insurance literacy. And, secondly, that the higher number of sources of health insurance information consulted would be related to higher mean HILM-NL scores. Both hypotheses were confirmed, indicating that the HILM-NL measures the intended construct of health insurance literacy. We were unable to perform a comparison with another instrument for measuring health insurance literacy since there are no such instruments available in the Netherlands.

The healthcare system in the United States differs in many ways from that of the Netherlands. An important difference is that the US government regulates healthcare to a lesser extent than the Dutch government. In the translation and adaption process, therefore, a number of questions from the original HILM instrument were adjusted so that they could apply to the Dutch healthcare system. We recommend that other researchers, who want to translate the original HILM instrument into another language, should first map out the systematic and cultural differences between the two healthcare systems and, as in the current study, use an expert panel with a wide variety of members.

We used a representative sample of the Dutch population, aged 18 and older, regarding sex and age from the Nivel Dutch Health Care Consumer Panel (DHCCP). However, in spite of this, the study was still unable to include specific groups of people, such as people who are illiterate. As a result, it is possible that the language level of the HILM-NL is slightly too high for a number of citizens in the Netherlands.

The concept of health insurance literacy has only recently received attention in the Netherlands. In other countries, more studies have already been carried out that focus on these skills among their citizens. For example, a study in the US showed that higher health insurance literacy skills are likely to lead to care being less often either delayed or foregone completely, owing to its cost [24]. This indicates that these skills influence the way citizens behave when they need care. In the Netherlands, follow-up research into the concept of health insurance literacy can also now be carried out using the translated and validated HILM-NL from the current study. We recommend determining whether such skills are related to the use of healthcare services, or the health insurance switching behaviour, among citizens in the Netherlands.

Altogether, the current study demonstrates that the HILM-NL is a reliable and valid instrument to measure health insurance literacy among citizens living in the Netherlands. It is possible, by using this instrument, to assess better how citizens in the Netherlands choose and use a health insurance policy, and the difficulties they face. It is a step forward in supporting these citizens better in making well-informed decisions about their health insurance.
